# Highlights of the Latest Advances in Research on CDK Inhibitors

**DOI:** 10.3390/cancers6042224

**Published:** 2014-10-27

**Authors:** Jonas Cicenas, Karthik Kalyan, Aleksandras Sorokinas, Asta Jatulyte, Deividas Valiunas, Algirdas Kaupinis, Mindaugas Valius

**Affiliations:** 1 CALIPHO Group, Swiss Institute of Bioinformatics, CMU-1, rue Michel Servet’ Geneva 4 CH-1211, Switzerland; 2 MAP Kinase Resource, Bern CH-3027, Switzerland; E-Mails: karthikms2004@gmail.com (K.K.); a.sorokinas@mapkinases.eu (A.S.); asta.jatulyte@gmail.com (A.J.); 3 Proteomics Centre, Vilnius University Institute of Biochemistry, Vilnius LT-08662, Lithuania; E-Mails: algirdas.kaupinis@gf.vu.lt (A.K.); mindaugas.valius@bchi.vu.lt (M.V.); 4 Systems Biomedicine Division and Department of Virology and Immunology, Haffkine Institute for Training Research and Testing, Mumbai 400 012, India; 5 Nature Research Centre, Vilnius LT-08412, Lithuania; E-Mail: deividas_valiunas@yahoo.com

**Keywords:** cancer, kinase, CDK, small molecule inhibitors

## Abstract

Uncontrolled proliferation is the hallmark of cancer and other proliferative disorders and abnormal cell cycle regulation is, therefore, common in these diseases. Cyclin-dependent kinases (CDKs) play a crucial role in the control of the cell cycle and proliferation. These kinases are frequently deregulated in various cancers, viral infections, neurodegenerative diseases, ischemia and some proliferative disorders. This led to a rigorous pursuit for small-molecule CDK inhibitors for therapeutic uses. Early efforts to block CDKs with nonselective CDK inhibitors led to little specificity and efficacy but apparent toxicity, but the recent advance of selective CDK inhibitors allowed the first successful efforts to target these kinases for the therapies of several diseases. Major ongoing efforts are to develop CDK inhibitors as monotherapies and rational combinations with chemotherapy and other targeted drugs.

## 1. Overview of CDKs and CDK Inhibitors

The cell cycle is a period between the successive divisions of a cell. During this period, the contents of the cell must be accurately replicated. The processes that permit the cell to divide are very precisely controlled by a multitude of enzymatic reactions amongst which the protein kinase-triggered protein phosphorylation plays a major role. In eukaryotes, there are four main stages/phases of cell cycle namely the Gap-1 (G1) phase, Synthesis (S) phase, Gap-2 (G2) and Mitosis (M) phases. An extended phase of Gap-1 phase is coined as Gap-0 (G0) phase or Resting phase [1].

**Figure 1:**
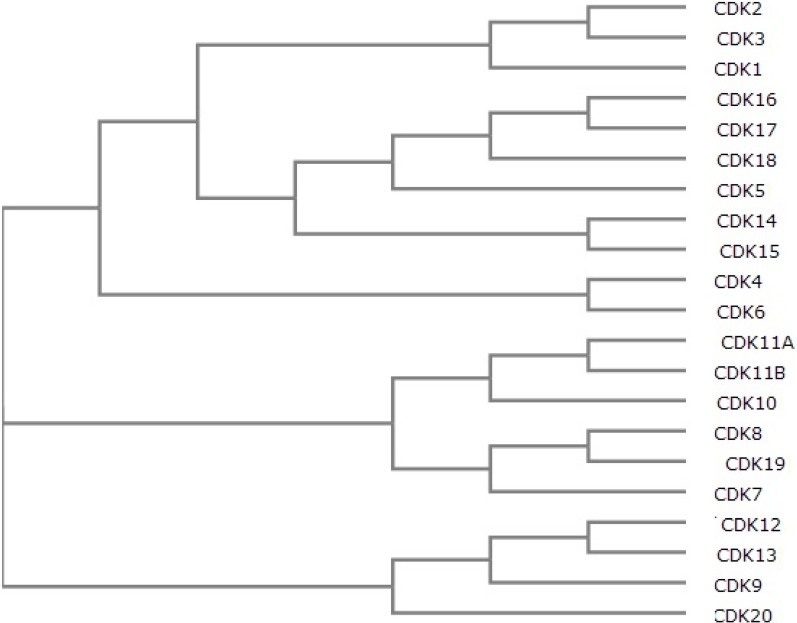
Phylogenetic tree of human CDKs (created using UniProt multiple alignment).

Cyclin-dependent kinases (CDKs) are members of subfamily of serine/threonine kinases which are found in both unicellular organisms such as yeast and multicellular organisms such as plants, humans and other mammals [1,2,3,4,5]. These kinases are dependent on cyclin-binding activity for their activation, thereby resulting in specific complex formation. In fission yeast Schizosaccharomyces pombe, the CDK1 gene encodes a 34 kDa protein serine/threonine kinase. The mutations in CDK1 cause the cell to halt at one of two distinct points: G1 or M phases [3]. In human genome, there are 21 genes that encode CDKs (Figure 1) and five genes encode more distant protein kinases termed as “CDK like kinases (CDKLs)” [6].

According to Malumbres and Barbacid [5], various CDKs activate the different stages of the cell cycle from G1 to mitosis. Each CDK/cyclin complex is responsible for transition or progression of a given phase within the cell cycle. Complexes of cyclin D in combination with CDKs 4 and 6; cyclin E in combination with CDK2 regulate the G0–G1 transition and the early phases of G1 via phosphorylation of Rb. The cyclin E/CDK2 complex is also involved in G1–S transition. CDK2 can also bind cyclin A for the entire progression of S-phase. The complexes of cyclin A in combination with CDK1 participate in the S–G2 and cyclin B in combination with CDK1 participates in the G2–M. CDK3 also seems to participate in the phosphorylation of Rb as it is highly related to CDK’s 1 and 2 and interacts with both cyclins A and E [7]. CDK5 is expressed in brain and are activated by proteins p35 and p39 which are not considered to be cyclins. CDK5 mostly acts in neural cells and is associated with activities such as cell survival, transcription, migration and membrane transportation [8]. CDK7, a constituent of both CDK-activating kinase (CAK) and general transcription factor TFIIH, acts upstream of cell cycle-regulatory CDKs and affects transcription [9]. The regulation of transcription is also performed by CDKs 8 [10] and 9 [11], which bind to cyclins C and T, respectively. Despite the lack of partner cyclin for CDK10, this kinase seems to play a role in the regulation of G2–M phase through the inhibition of Ets2 transactivation [12]. The CDK11 can be involved in mRNA splicing upon binding to cyclin L [13] as well as repress proliferation upon its interaction with cyclin D [14]. CDK 12 and 13 apparently bind cyclin K and subsequently maintain the genome stability, primarily by phosphorylating serine 2 in the C-terminal domain of RNA polymerase II [15]. Cyclin Y in a complex with 14-3-3 proteins activates CDK14, which is important in embryonic patterning [16]. CDK15 acts like an antiapoptotic protein by inducing phosphorylation of surviving at threonine 34 [17]. No cyclin-like, or other protein interaction is known for this kinase to date.

Most CDKs are known to play a role in different types of human cancers [4]. CDK1 is known to have a diagnostic value in esophageal and breast cancers, while the expression of CDK2 or its activity seems to have been utilized towards the prognosis of breast, ovarian and oral cancers. In other types of cancer such as the ovarian, urinary bladder, endometrial or oral, CDK4 expression is impaired. CDK5 is known to play a role in the lung cancer, while CDK6 is misexpressed in oral cancer. The polymorphisms of CDK7 are known to effect breast cancer.

Abnormalities in CDK characteristics such as its expression, activity and regulation have also been found in pathological conditions such as viral infections, neurodegenerative disorders and proliferative diseases *etc.* That led to a rigorous search for small-molecule CDK inhibitors for the therapeutic purposes.

The first CDK inhibitor was 2-hydroxyethylamino-6-benzylamino-9-methylpurine, which was discovered by Vesely and Meijer [18]. It was later renamed olomoucine, after Olomouc, the town of Vesely’s university in the Czech Republic. It was the first inhibitor, which had the selectivity for CDKs (IC_50_ = 3–7 μM) and, to a lesser extent, for MAP kinases (IC_50_ = 25 μM). It was found to target the ATP-binding pocket of CDKs and inhibit them by competing with ATP binding.

The next inhibitor, specific for CDKs was 2-(*R*)-(1-ethyl-2-hydroxyethylamino)-6-benzylamino-9-isopropylpurine, which was later renamed roscovitine (after Roscoff, where the lab which discovered the compound was located) for convenience [18]. (*R*)-Roscovitine is also known by the names of CYC-202 and seliciclib. It inhibits CDK1, CDK2, CDK5 and CDK7 (IC50 = 0.2–0.5 μM) but is a poor inhibitor for CDK4 and CDK6 (IC50 > 100 μM) [4]. Roscovitine was also tested in Phase I clinical trial for patients with solid tumors [19]. Results showed lack of activity, but disease stabilization was detected in eight out of 25 patients. Another Phase I trial for patients with hepatocellular carcinoma, showed partial response in one patient and tumor stabilization in six out of 25 patients involved [20].

The discovery of olomoucine and roscovitine and their co-crystal structures with CDK2 inspired the whole field of discovery of purine analogue inhibitors. That ultimately led to the identification of purvalanols which were very potent highly selective. Purvalanol A is an inhibitor selective for CDK2 (IC_50_ = 4–70 nM) and CDK5 (IC_50_ = 75 nM), but less selective toward CDK4 (IC_50_ = 850 nM) [4]. Purvalanol B is a selective for CDK1 (IC_50_ = 6 nM), CDK2 (IC_50_ = 6–9 nM), CDK5 (IC_50_ = 6 nM), but unselective towards CDK 4/6 or other kinases (IC_50_ > 10000).

**Figure 2:**
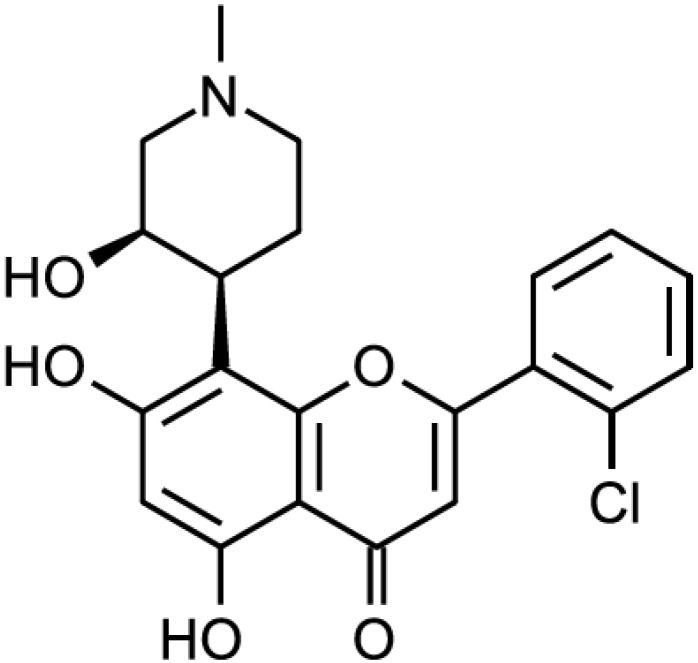
Flavopiridol.

Another type of CDK inhibitors discovered early is the flavonoids. The first discovered and most prominent of those is flavopiridol, also variously known as L-868275, HMR-1275, alvocidib or NSC-649890 (Figure 2). It is a broad-range CDK inhibitor. It inhibits CDK9 at IC_50_ value of 8nM and CDK1, CDK2, CDK4 and CDK7 in an IC_50_ value range of 0.04–0.4 μM. It less effectively inhibits epidermal growth factor receptor (IC_50_ = 21 μM) and protein kinase A (IC_50_ = 122 μM) [4]. Flavopiridol was the first CDK inhibitor used in human clinical trials. Several Phase I clinical trials showed that flavopiridol had an antitumor effect as single agent [20,21,22] or in combination with chemotherapeutic drugs [23,24,25,26,27,28,29]. Several Phase II trials were also performed, showing high response rates in chronic myeloid leukemia [30] and medium responses in pancreatic cancer [31].

Yet another compound fascaplysin, originally isolated from the sponge *Fascaplysinopsis bergquist* [32,33], is a kinase inhibitor selective for CDK4 (IC_50_ = 0.35 μM) and CDK6 (IC_50_ = 3.4 μM) and not selective for the other CDKs or other kinases. (IC_50_~500 μM) [4].

Dioxobenzothiazoles, studied for their antifungal activities, were also found to be selective and potent inhibitors of CDKs. Namely 5-arylamino-2-methyl-4,7-dioxobenzothiazoles showed incredible selectivity towards CDK4 (IC_50_ = 6–7μM). They were also quite cytotoxic against various cancer cells (IC_50_ = 0.2–3.6 μg/mL). One of these compounds was commercialized under the name of ryuvidine [34].

To date, more than 20 different CDK small molecule inhibitors have been developed [4,35], which can be subdivided into two main groups: broad-range inhibitors (such as above mentioned flavopiridol, olomoucine and roscovitine) and specific inhibitors (such as above mentioned purvalanols, fascaplysin and ryuvidine) (Table 1).

**Table 1:** List of CDK inhibitors.

Inhibitor	Alternative Names	Kinases Inhibited	In Clinical Development Yes/No	Refs.
3α-Amino-5α-androstane		CDK5	No	[36]
7x		CDK4, ARK5	No	[37]
AG-024322		CDK1, CDK2, CDK4	Yes	[4]
AMG 925		CDK4, FLT3	No	[38]
AT7519		CDK1, CDK2	Yes	[39]
AZD5438		CDK1, CDK2, CDK4, CDK5, CDK6, CDK9	Yes	[4]
BAY 1000394		CDK1, CDK2, CDK3, CDK4, CDK7, CDK9	No	[40]
BML-259		CDK2, CDK5	No	[4]
Compound **1**		CDK4, ABL, FGFR1, FYN, KDR, LCK, LYN, SRC	No	[41]
Compound **530**		CDK4, CDK4	No	[42]
CR8		CDK2, CDK5	No	[43]
Dinaciclib	MK-7965, SCH 727965	CDK1, CDK2, CDK5, CDK9	Yes	[44]
F07#13		CDK2, CDK9	No	[45]
Fascaplysin		CDK4, CDK6	No	[4]
Flavopiridol	L-868275, HMR-1275, alvocidib, NSC-649890	CDK1, CDK2, CDK4, CDK7	Yes	[4]
Kenpaullone	NSC 664704, 9-bromopaullone	CDK1, CDK2, CDK5	No	[4]
LY2835219	abemaciclib	CDK4, CDK6	Yes	[46]
NBI1		CDK2	No	[47]
NU2058		CDK1, CDK2	No	[4]
Olomoucine		CDK1, CDK2, CDK5	No	[4]
P276-00		CDK1	Yes	[4]
PD-0332991		CDK4, CDK6	Yes	[4]
PHA-793887		CDK1, CDK2, CDK4	Yes (Stopped)	[48]
Purvalanol A/B		CDK1, CDK2, CDK5	No	[4]
R547	Ro-4584820	CDK1, CDK2, CDK4	Yes	[4]
RGB-286638		Pan-CDK	No	[49]
Roscovitine	CY-202, (*R*)-roscovitine, seliciclib	CDK2, CDK5	Yes	[4]
Ryuvidine		CDK4	No	[4]
SNS-032	BMS-387032	CDK2, CDK7, CDK9	Yes	[4]
SU 9516		CDK1, CDK2	No	[4]
VMY-1-101		CDK2, CDK5, CDK7	No	[50]
VMY-1-103		CDK2, CDK5, CDK7	No	[50]

## 2. Advances in Preclinical Studies

One of the interesting fields in CDK inhibitor research in recent years was the advance of ATP-noncompetitive inhibitors. Lo *et al.* identified an ATP-noncompetitive compound, using time-resolved fluorescence resonance energy transfer assay (TR-FRET) by screening more than 250,000 compounds and determining their IC_50_ values against CDK4 [41]. Three compounds were found to have an IC_50_ ratio below 2.5 μM. Compound **1** was the further tested for the specificity and it was found that is highly selective for CDK4 *versus* the other 34 serine/threonine kinases tested. However, it was also potent against a number of tyrosine kinases tested, such as ABL, FGFR1, FYN, KDR, LCK, LYN, and SRC. Another ATP-noncompetitive compound, an all D-amino acid hexapeptide, termed NBI1, which interferes with the formation of the CDK2/cyclin A complex, was found to induce apoptosis and inhibit proliferation of tumor cell lines [47]. Moreover, it was shown that NBI1 sensitizes erlotinib-resistant tumor cells to the treatment and erlotinib-sensitive cells to the smaller dose of erlotininb [51]. Premnath *et al.* used replacement with partial ligand alternatives through computational enrichment (REPLACE) approach to design ATP-noncompetitive CDK inhibitors [42]. Inhibitory peptides were used as basis, and then converted to a less peptidic inhibitor. The 3,4-diethoxy analog (compound **530**) was found to be the most potent against both CDK2 (IC_50_ = 5.2 μM) and CDK4 (IC_50_ = 3 μM). Van Duyne *et al.* designed a new CDK9 and CDK2 inhibitor, using structure-based analysis of cyclin/CDK complexes as well as blocking peptides [45]. After screening 52 compounds, one of them, named F07#13 had an IC_50_ of 0.12 μM towards CDK2 and CDK9, which it achieved by disrupting interactions between CDK2/Cyclin E and CDK9/Cyclin T. Moreover, it inhibited HIV-1 viral replication in humanized mouse models. Bioluminescence resonance energy transfer (BRET)-based screening assay has also been used to identify 3α-amino-5α-androstane as an inhibitor of CDK5/p25 interaction, and thus CDK5 kinase activity (IC_50_ = 6 μM) [36].

Another interesting approach is a design of fluorescent CDK inhibitors. Yenugonda *et al.* using rational drug design, designed and fluorescent CDK inhibitors VMY-1-101 and VMY-1-103, which are based on a purvalanol B [50]. They were found to be potent inhibitors of CDK2, CDK5 and CDK7 and had an anti-proliferative activity (IC_50_ = 4 μM or 10–19 μM for VMY-1-103 or VMY-1-101, respectively) against breast cancer cell lines. In addition these inhibitors were easily traceable, therefore intracellular localization of the compounds could be tracked using confocal microscopy.

Recently, there were a lot of efforts studying both long-known as well as newer CDK inhibitors in animal cancer models. AMG 925, a FLT3/CDK4 dual inhibitor was found to inhibit growth of subcutaneous MOLM13 xenograft tumors and systemic MOLM13-Luc xenograft tumors in nude mice, a disease model for acute myeloid leukemia [38]. A new CDK4/ARK5 inhibitor 8-cyclopentyl-2-[4-(4-methylpiperazin-1-yl)-phenylamino]-7-oxo-7,8-dihydropyrido[2,3-d]pyrimidine-6-carbonitrile, also known as 7x, was shown to lead to a dose-dependent inhibition of tumor growth and reduction in tumor weight over a 21 day period in nude mice breast cancer model [37]. PD-0332991, a CDK4/6 inhibitor prolonged survival in the Ink4a-ARF deficient brainstem glioma model mice (42 days *vs.* 47 days, *p* = 0.033) [52]. Hayashi *et al.* found that flavopiridol in combination with temozolomide suppressed tumor growth in subcutaneous glioma nude mouse xenografts (*p* < 0.05) [53]. RGB-286638, a broad-range CDK inhibitor, was found to significantly suppress multiple myeloma tumor growth in SCID mice, with more than 80% tumor growth inhibition [49]. BAY 1000394, a pan-CDK inhibitor, was also effective in a xenograft model of mammary tumor in nude mice [40]. Martin *et al.* found that radiation-induced salivary gland dysfunction can be prevented by roscovitine pretreatment, which might prevent the side effects of radiation therapy in tumor surrounding normal tissues [54]. Roscovitine and its derivative CR8 were also found to inhibit renal cystogenesis and hepatic cystogenesis in Pkd1-conditional knockout mice [55]. AZD5438, a CDK1, CDK2, and CDK9 inhibitor, was found to enhance the radiosensitivity of non-small cell lung carcinoma cells in athymic nude mice [39]. The CDK4 inhibitor P276, in combination with gemcitabine, inhibited tumor growth and angiogenesis of pancreatic cancer cell xenografts in nude mice [56]. CDK inhibitors, targeting neuronal functions we also studied in animal models. Yang *et al.* studied roscovitine in a rat model of chronic compression of dorsal root ganglion and found that that intrathecal administration of roscovitine alleviates pain by downregulating the expression of NR2A [57]. CR8 a second-generation analog of roscovitine, was shown to provide neuroprotection in experimental traumatic brain injury by reducing cortical, hippocampal, and thalamic neuronal loss and cortical microglial and astrocyte activation [43,58].All the inhibitor information mentioned in this section is summarized in Table 2.

**Table 2:** List of CDK inhibitors described in preclinical study section.

Inhibitor	Kinases Inhibited	Tested	Disease(s)	Refs.
Compound **1**	CDK4, ABL, FGFR1, FYN, KDR, LCK, LYN, SRC	*In vitro*	n.a.	[41]
NBI1	CDK2	*In vitro*, cell lines HCT116, HT29, T98G, A2780, MDA-MB-468, SKBr3, MCF-7, A549, Jurkat, HL60 and Saos-2	colon carcinoma, glioblastoma, ovarian carcinoma, breast carcinoma, acute myeloid leukemia, lung carcinoma, osteosarcoma	[47,51]
Compound **530**	CDK2, CDK4	*In vitro*	n.a.	[42]
F07#13	CDK2, CDK9	*In vitro*, mouse models	AIDS	[45]
3α-Amino-5α-androstane	CDK5	*In vitro*, Saccharomyces cerevisiae	n.a.	[36]
VMY-1-101	CDK2, CDK5, CDK7	*In vitro*, cell lines MDA-MB-231, MCF-7	breast carcinoma	[50]
VMY-1-103	CDK2, CDK5, CDK7	*In vitro*, cell lines MDA-MB-231, MCF-7	breast carcinoma	[50]
AMG 925	CDK4, FLT3	xenograft mouse model	acute myeloid leukemia	[38]
7x	CDK4, ARK5	*In vitro*, panel of human tumor cell lines, xenograft mouse model	various human cancers (cell lines), breast carcinoma (mouse model)	[37]
PD 0332991	CDK4, CDK6	mouse model	brainstem glioma	[52]
Flavopiridol	CDK1, CDK2, CDK4, CDK7	xenograft mouse model	glioma	[53]
RGB-286638	pan-CDK	xenograft mouse model	multiple myeloma	[49]
BAY 1000394	CDK1, CDK2, CDK3, CDK4, CDK7, CDK9	mouse model	breast carcinoma	[40]
Roscovitine	CDK2, CDK5	mouse model	radiation-induced salivary gland dysfunction, renal and hepatic cystogenesis, pain	[54,55,57]
CR8	CDK2, CDK5	mouse model	renal and hepatic cystogenesis, traumatic brain injury	[43,55,58]
AZD5438	CDK1, CDK2	xenograft mouse model	non-small cell lung carcinoma	[39]
P276	CDK4	xenograft mouse model	pancreatic carcinoma	[56]

## 3. Advances in clinical studies

Flavopiridol was still popular in clinical trials of recent several years. A Phase I study of flavopirirdol as a single agent was performed on relapsed myeloma after at least two prior treatments [59]. Fifteen patients were treated and only one of them had marginal response (a decrease in his monoclonal protein >50%). Side effects included significant diarrhea, cytopenias and transaminase elevation. Another Phase I trial involved flavopiridol in combination with doxorubicin in advanced sarcomas [60]. 28 patients were involved and the combination of flavopiridol and doxorubicin achieved a considerable disease control, with 68% (19/28) achieving partial response or stable disease. Main dose-limiting toxicities were neutropenia, leukopenia, and febrile neutropenia. Yet another Phase I clinical trial was dealing with the combination of flavopiridol and imatinib mesylate in patients with Bcr-Abl+ chronic myelogenous leukemia [61]. Of 21 patients enrolled in a study, five patients responded, including four sustained responses (for 2, 4, 15 and 30 months, respectively) and four patients had stable disease. No clinically significant pharmacokinetic interactions between imatinib and flavopiridol were observed. A Phase II trial was performed on the combination of flavopiridol with cisplatin in platin-resistant ovarian and primary peritoneal carcinoma [62]. Of 40 platin-resistant patients seven patients achieved a confirmed response and ten patients maintained stable disease. Four of five platin-sensitive patients responded, but the cohort was closed. Main toxicity symptoms included neutropenia, nausea, vomiting, fatigue, thrombosis and anemia. Of nine high-risk chronic lymphocytic leukemia patients treated with flavopiridol, cyclophosphamide and rituximab combination, three achieved complete response, four-partial response, one had stable disease, and one had progressive disease [63]. The overall response rate of 46% was observed in a cohort of 112 patients with refractory or relapsed chronic lymphocytic leukemia, treated with flavopiridol as a single agent [64]. Another study of 116 patients, divided into two categories (≥70 years and <70 years) for comparison, showed feasibility of flavopiridol as single agent in elderly patients (response rates (43% *vs.* 47%, respectively)) [65]. Phase I trial of bortezomib and flavopiridol in patients with recurrent or refractory B-cell neoplasms, showed overall response rate of 44% [66]. Another Phase I trial of bortezomib and flavopiridol in patients with recurrent or refractory indolent B-cell neoplasms performed by the same group, showed 33% overall response rate [67].

**Figure 3:**
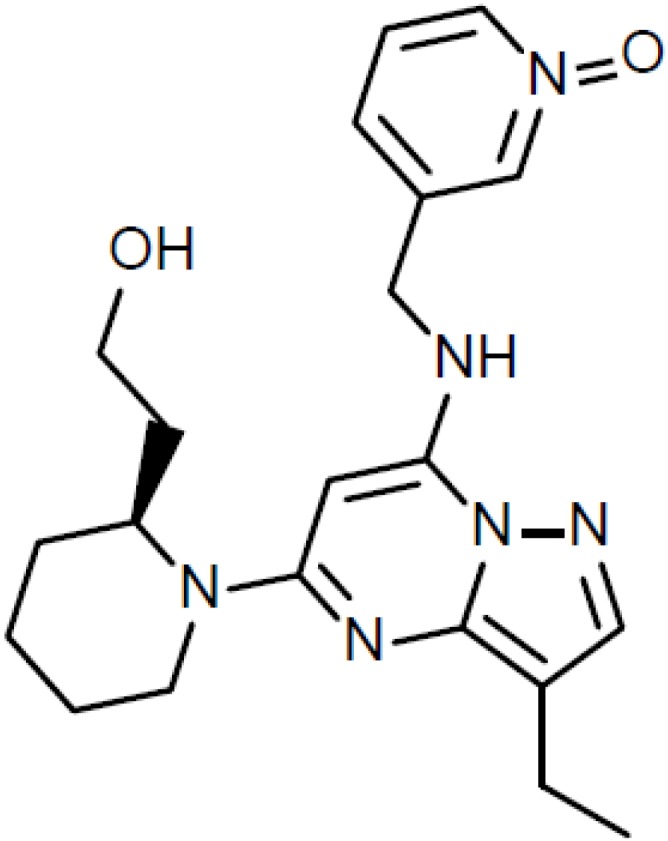
Dinaciclib.

One of the most popular CDK inhibitor in clinical trials in the recent years was dinaciclib (MK-7965, SCH 727965) (Figure 3), the inhibitor of CDK1, CDK2, CDK5, and CDK9. A Phase I trial on the effect of dinaciclib in combination with aprepitant was performed in patients with advanced malignancies [44]. Aprepitant is used for the prevention of chemotherapy-induced nausea and vomiting, is known as an inhibitor and inducer of CYP3A4, which metabolizes dinaciclib. Coadministration of dinaciclib with aprepitant resulted in no clinically significant effect on the pharmacokinetics and did not alter the safety profile of dinaciclib. The first Phase I clinical trial on dinaciclib as a single agent was performed on patients with advanced malignancies [68]. Forty-eight patients with various solid tumors were treated and 10 of them achieved prolonged stable disease for at least four treatment cycles. Adverse effects were mild, the most common being nausea, anemia, decreased appetite and fatigue. A phase II multi-center study of dinaciclib for relapsed and/or refractory AML was performed on 20 patients [69]. Temporary decrease in peripheral blood and/or bone marrow blasts was observed in 60% of patients. Four of 13 (31%) patients with circulating blasts had >50% decrease and 6 (46%) >80% decrease in the absolute blast count within 1–8 days of the first dinaciclib dose. Toxicities included diarrhea, fatigue, transaminitis, and manifestations of tumor lysis syndrome, with one patient who deceased of acute renal failure. Another Phase II study was performed of dinaciclib *versus* erlotinib in patients with non-small cell lung cancer [70]. Unfortunately, it was found that dinaciclib was not successful as monotherapy in non-small cell lung cancer. Most common toxicities included neutropenia, leukopenia, vomiting, and diarrhea. Yet another Phase II study was performed on dinaciclib *versus* capecitabine in patients with advanced breast cancer [71]. Dinaciclib treatment demonstrated antitumor activity in two of seven patients with ER-positive and ERBB 2-negative metastatic breast cancer, however efficacy was not superior to capecitabine (*p* = 0.991). Toxicities included neutropenia, leukopenia, increase in aspartate aminotransferase, and febrile neutropenia. Phase I nonrandomized dose-escalation trial was performed, where patients with relapsed or refractory chronic lymphocytic leukemia were treated with dinaciclib and rituximab [72]. Four out of six patients achieved stable disease, and one patient achieved complete response. Drug-related adverse events were mostly hematological, digestive and metabolic and no dose-limiting toxicities were observed. Dinaciclib was also moved into Phase III development for refractory chronic lymphocytic leukemia [73]. Phase I/II clinical trial Dinaciclib in patients with relapsed multiple myeloma showed promise as single agent [74]. The overall confirmed response rate was 3 of 27 (11%). Adverse effects included leukopenia, thrombocytopenia, gastrointestinal symptoms, alopecia, and fatigue.

**Figure 4:**
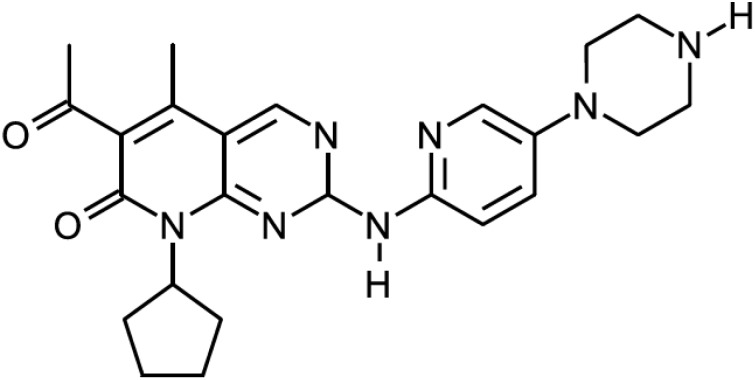
PD 0332991.

PD 0332991, a CDK4/6 inhibitor (Figure 4), was used in a Phase I clinical trial in patients with advanced cancer [75]. Out of 37 patients 10 (27%) had stable disease for ≥4 cycles of whom six (16%) had prolonged benefit (≥10 cycles). Main toxicities included neutropenia, anemia, leukopenia, fatigue, nausea, and diarrhea. A Phase II trial in patients with advanced CDK4-amplified well-differentiated or dedifferentiated liposarcoma was also performed [76]. Of 29 patients, 19 (66%) were progression free at 12 weeks, which exceeded expected rate of 40%. Main toxicities included anemia, thrombocytopenia, neutropenia and febrile neutropenia. A Phase II trial of PD0332991 was performed in 37 patients with advanced breast cancer (mostly ER-positive ERBB2-negative), pretreated with chemotherapy [77]. Two of patients had partial response, and 18 had disease stabilization as best response. Drug was well tolerated; toxicities were uncomplicated and included neutropenia, leucopenia, lymphopenia, and thrombocytopenia. Encouraging data led to the Phase II study expansion where patients were randomized to combination therapy compared with letrozole alone as first-line treatment for breast cancer, which was designed in two parts [78]. In first part (66 patients), patients were clinically selected based on ER-positive HER2-negative breast cancer. Partial response rate and disease stabilization were higher for the letrozole and PD 0332991 combination (52% *versus* 32% and 35% *versus* 25%, respectively). This significant improvement led to the FDA breakthrough therapy designation in April 2013. In the second part (61 patients), additional eligibility criteria included cyclin D1 gene amplification and/or p16 loss. There was no correlation found between these markers and outcomes, however data is not final yet. Based on these results a Phase III trial of PD-0332991 in estrogen receptor ER-positive breast cancer is planned [73].

Another CDK4/6 inhibitor, LY2835219 (abemaciclib), was shown to be beneficial as monotherapy for patients with metastatic breast cancer, mainly for those with ER/PgR—positive disease [46]. Of 47 patients, 9 (19%) had a partial response, 24 (51%) had stable disease, 11 (23%) experienced disease progression, and three could not be evaluated for a response. The most frequent toxicities included diarrhea, nausea, fatigue, neutropenia, vomiting, and decreased platelet and white-blood cell counts.

Unfortunate fate awaited the CDK1, CDK2 and CDK4 inhibitor PHA-793887 in a Phase I clinical study [48]. It was shown that PHA-793887 induces severe, dose-related hepatic toxicity, and any further clinical development was stopped. All the inhibitor information, mentioned in this section is summarized in Table 3.

## 4. Perspectives

Recent developments in the small-molecule CDK inhibitor field led to quite a number of compounds with a different range of the inhibited targets, anticancer potencies both *in vitro* and *in vivo* and modes of molecular action. However, there is still much room for improvement. First of all, the specificity of the inhibitors, which inhibit close isoforms, is not fully achieved. Especially that is relevant for CDK5, which is involved in neurodegenerative diseases and CDK9, involved in viral replication. Structure and bioinformatics—based approaches should help tackle this problem. On the other hand it is still unclear which CDK or range of CDKs should be targeted in a clinical setup. Clinical (and maybe also preclinical) studies should help to determine whether it is better to use highly selective or rather broad range CDK inhibitors as anticancer therapy.

It also seems that combination therapies with CDK inhibitors are more promising than monotherapies, therefore more chemotherapeutic agents should be evaluated in combination with CDK inhibitors. Other targeted drugs seem to work nicely in combination; therefore this field of clinical research should also be further pursued.

**Table 3:** List of CDK inhibitors described in clinical study section.

Treatment	Clinical Trial Phase	Disease(s)	Response Rate	Adverse Effects	Refs.
Flavopiridol	Phase I	relapsed myeloma	1/50 (2%)	diarrhea, cytopenias, transaminase elevation	[59]
Flavopiridol in combination with doxorubicin	Phase I	advanced sarcomas	19/28 (68%)	neutropenia, leukopenia, febrile neutropenia	[60]
Flavopiridol in combination with imatinib mesylate	Phase I	Bcr-Abl + chronic myelogenous leukemia	5/21 (24%)	anemia, leukopenia, lymphopenia, thrombocytopenia	[61]
Flavopiridol in combination with cisplatin	Phase II	platin-resistant ovarian and primary peritoneal carcinoma	17/40 (43%) platin-resistant patients; 4/5 (80%) platin-sensitive patients	neutropenia, nausea, vomiting, fatigue, thrombosis, anemia	[62]
Flavopiridol in combination with cyclophosphamide and rituximab	Phase I	chronic lymphocytic leukemia	7/9 (78%)	fatigue, electrolyte disturbances, diarrhea, abdominal discomfort, nausea/vomiting, liver dysfunction, anemia, leukopenia, neutropenia, thrombocytopenia	[63]
Flavopiridol	Phase I/II	chronic lymphocytic leukemia	112/52 (46%)	n.a.	[64]
Flavopiridol	?	chronic lymphocytic leukemia	41/95 (43%) ≥70 years old; 10/21 (47%) <70 years old	tumor lysis syndrome, cytokine release syndrome, neutropenia, diarrhea, fatigue	[65]
Flavopiridol in combination with bortezomib	Phase I	refractory B-cell neoplasms	7/16 (44%)	neutropenia, lymphopenia, and thrombocytopenia	[66]
Flavopiridol in combination with bortezomib	Phase I	Refractory indolent B-cell neoplasms	13/39 (33%)	leukopenia, lymphopenia, neutropenia, thrombocytopenia, diarrhea, fatigue, sensory neuropathy	[67]
Dinaciclib in combination with aprepitant	Phase I	advanced malignancies	n.a.	no change in safety profile of dinaciclib	[44]
Dinaciclib	Phase I	advanced malignancies	10/48 (21%)	nausea, anemia, decreased appetite and fatigue	[68]
Dinaciclib	Phase I	relapsed and/or refractory acute myeloid leukemia	12/20 (60%)	diarrhea, fatigue, transaminitis, manifestations of tumor lysis syndrome; one patient deceased of acute renal failure	[69]
Dinaciclib *vs.* erlotinib	Phase II	non-small cell lung cancer	Not successful	neutropenia, leukopenia, vomiting, diarrhea	[70]
Dinaciclib *vs.* capecitabine	Phase II	advanced breast cancer	2/7 (29%) (not superior to capecitabine)	neutropenia, leukopenia, increase in aspartate aminotransferase, febrile neutropenia	[71]
Dinaciclib *vs.* capecitabine	Phase I	chronic lymphocytic leukemia	5/6 (83%)	hematological, digestive and metabolic; no dose-limiting toxicities	[72]
Dinaciclib	Phase I/II	relapsed multiple myeloma	3/27 (11%)	leukopenia, thrombocytopenia, gastrointestinal symptoms, alopecia, fatigue	[74]
PD 0332991	Phase I	advanced cancer	10/37 (27%)	neutropenia, anemia, leukopenia, fatigue, nausea, diarrhea	[75]
PD 0332991	Phase I	advanced CDK4-amplified well-differentiated or dedifferentiated liposarcoma	19/29 (66%)	anemia, thrombocytopenia, neutropenia, febrile neutropenia	[76]
PD 0332991	Phase II	advanced breast cancer	20/37 (%)	neutropenia, leucopenia, lymphopenia, thrombocytopenia	[77]
PD 0332991 in combination with letrozole *vs.* letrozole alone	Phase II	advanced breast cancer	87% *vs.* 57% (66 patients)	neutropenia, leukopenia, and fatigue	[78]
LY2835219	Phase I	metastatic breast cancer	33/47 (70%)	diarrhea, nausea, fatigue, neutropenia, vomiting, decreased platelet and white-blood cell counts	[46]
PHA-793887	Phase I	solid tumors	n.a.	severe, dose-related hepatic toxicity	[48]
